# Prevalence and genotype distribution of hepatitis delta virus among chronic hepatitis B carriers in Central Vietnam

**DOI:** 10.1371/journal.pone.0175304

**Published:** 2017-04-12

**Authors:** Hung Minh Nguyen, Bui Tien Sy, Nguyen Thanh Trung, Nghiem Xuan Hoan, Heiner Wedemeyer, Thirumalaisamy P. Velavan, C-Thomas Bock

**Affiliations:** 1 Center for Molecular Biology, Institute of Research and Development, Duy Tan University, Da Nang, Vietnam; 2 Department of Molecular Biology, 108 Military Central Hospital, Hanoi, Vietnam; 3 Institute of Tropical Medicine, University of Tübingen, Tübingen, Germany; 4 German Center for Infection Research, Department for Gastroenterology, Hepatology, and Endocrinology, Medical School Hannover, Hannover, Germany; 5 Vietnamese-German Center for Medical Research, Hanoi, Vietnam; 6 Department of Infectious Diseases, Robert Koch Institute, Berlin, Germany; Singapore Institute for Clinical Sciences, SINGAPORE

## Abstract

Hepatitis D virus (HDV) infection plays an important role in liver diseases. However, the molecular epidemiology and impact of HDV infection in chronic hepatitis B (CHB) remain uncertain in Vietnam. This cross-sectional study aimed to investigate the prevalence and genotype distribution of HDV among HBsAg-positive patients in Central Vietnam. 250 CHB patients were tested for HDV using newly established HDV-specific RT-PCR techniques. HDV genotypes were determined by direct sequencing. Of the 250 patients 25 (10%) had detectable copies of HDV viral RNA. HDV-2 was predominant (20/25; 80%) followed by HDV-1 (5/25; 20%). Proven HDV genotypes share the Asian nomenclature. Chronic hepatitis B patients with concomitant HDV-1 showed higher HBV loads as compared to HDV-2 infected patients [median log10 (HBV-DNA copies/ml): 8.5 vs. 4.4, P = 0.036]. Our findings indicate that HDV infection is highly prevalent and HDV-2 is predominant in Central Vietnam. The data will add new information to the management of HBsAg-positive patients in a highly HBV endemic region to in- or exclude HDV infection in terms of diagnostic and treatment options.

## Introduction

Hepatitis D, caused by the hepatitis Delta virus (HDV) is a unique RNA pathogen, which needs hepatitis B virus (HBV) envelope proteins to infect the hepatocytes. HBV-HDV coinfection can accelerate the liver diseases [1–4]. Among 250 million chronic HBsAg carriers worldwide [5], approximately 12.5 million individuals had been coinfected with HDV [6].

HDV is a small single-stranded negative-sense circular RNA virus which encompasses a spherical “HDAg and HBsAg-hybrid” particle of ~36 nm in diameter, and an outer envelope containing hepatitis B surface antigens (HBsAg) [7, 8]. The HDV genome consists of approx. 1680 nucleotides covered by approximately 200 hepatitis D antigen molecules (HDAg) [2, 9]. HDV needs the presence of HBV envelope proteins for its assembly, propagation, and transmission to initiate new rounds of infection [6, 10]. However, HDV can replicate within non HBV infected hepatocytes since no helper function for HDV RNA synthesis and RNP assembly is required by HBV [10, 11]. The open reading frame of HDV encodes unique delta antigens, the small and the large isoforms of S-HDAg and L-HDAg. The S-HDAg is responsible for HDV replication initiation, whereas the L-HDAg is required for viral packaging [10, 12]. HDV can be acquired either as a coinfection with acute hepatitis B or as a super infection especially in chronically HBV infected individuals [13].

HDV viruses have been separated into at least eight major clades based on their genome diversity, with specific geographic distribution [6, 14]. HDV-1 is the frequently occurring clade and is distributed among Europe, Middle East, America and North Africa [15–18]. HDV-2 to HDV-8 occur regionally [19]. For instance, HDV-2 prevails in countries like Japan [20], Taiwan [21, 22] and Russia [23]. HDV-3 which is a most diverged genotype is exclusively found in South America [18, 24, 25]. HDV-4 is reported across Japan and Taiwan [21]; whereas HDV-5 to HDV-8 are described in Africa [26, 27].

The distribution of HDV varies with different geographical regions with a higher incidence in Middle East, Mediterranean, Amazonas, African, and in Asian countries [6, 14, 28]. Vietnam is a South-East Asian country with a high prevalence of HBV infection and approximately 10–20% of general populations live with chronic hepatitis B [29, 30]. High prevalence of HDV is reported in HBsAg positive patients in Northern provinces of Vietnam, especially in patients with acute hepatitis B with up to 43% [31]. The previous study reported that 15% of HBV patients were positive for HDV-RNA and HDV-2 is predominant in this specific region [31]. In contrast, another studies conducted in two rural districts from Northern Vietnam reported a low HDV prevalence (1%) in HBsAg positive individuals [32]. Although studies have shown considerable proportion of HDV-HBV coinfection in Northern provinces, no studies exist till date on the prevalence of HDV-HBV coinfection in Central and South provinces of Vietnam. Therefore, this study aimed to investigate the prevalence and genotype distribution of HDV in HBsAg-positive patients in Central Vietnam. Furthermore, HDV genotypes were correlated with clinical aspects in HBV-HDV coinfected patients.

## Materials and methods

### Study subjects

A total of 250 chronic hepatitis B patients were recruited for this cross-sectional study from March to June 2015. The study subjects visited for blood checking or admitted for the treatment in the Hoan My hospital, Da Nang, Vietnam. These patients were from three South Central provinces, namely Da Nang, Quang Nam, and Quang Ngai provinces.

The inclusion criteria were HBV patients positive for HBsAg and negative for anti-HCV and anti-HIV antibodies as determined by ELISA assays (Diagnostic automation/Cortez Diagnostics, Inc., Woodland Hills, California, USA). Patients with other relevant liver diseases such as autoimmune hepatitis, alcoholic or toxic liver disease, and hemochromatosis were excluded from the study. All HBV patients were treatment naïve. All patients did not present cirrhosis and/or liver cancer. The clinical parameters were collected, including ALT, AST, total bilirubin, direct bilirubin. Five ml of venous blood were collected from all participants at the time of visit or admission. Serum or plasma was separated from blood and used for biochemical and other laboratory routine procedures. Samples were stored at -80°C until further use.

### Ethics statement

Informed written consent was obtained after detailed explanation of the study at the time of sampling from all participants or from their parents if subjects were less than 18 years. The study was approved by the institutional review board of the Hoan My Hospital, Da Nang, Vietnam and from Duy Tan University, Da Nang, Vietnam.

### Viral nucleic acid extraction and cDNA synthesis

Nucleic acid (DNA and RNA) were extracted from 200 μL sera using High Pure Viral Nucleic Acid kit (Roche Diagnostics GmbH, Mannheim, Germany) following the manufacturer’s instruction and stored until use in aliquots at -80°C. Synthesis of cDNA was performed using First Strand cDNA Synthesis Kit for RT-PCR (Roche GmbH, Mannheim, Germany) following the manufacturer’s instructions.

### Molecular detection and genotyping of HDV

HDV-specific nested PCRs were performed with reverse transcribed cDNA samples as template using four highly conserved primer pairs representing all eight HDV genotypes as previously described [31]. HDV nomenclature was according recent reports [26, 33]. The first round of the PCR amplification was performed with 100 ng of cDNA using the FastStart PCR Master Kit (Roche Diagnostics GmbH, Mannheim, Germany) and primer pairs HDV57-F and HDV60-R (nucleotide 299 to 770 as referred to NC001653). The amplicons from first round were used as templates for the subsequent second PCR using primer pairs HDV48-F and HDV54-R targeting specific regions in the HDV genome. The details of primer pairs employed and specific regions along with necessary conditions are described in Table 1.

**Table 1:** Primers used for HDV detection.

Primers	Sequence (5’- 3’)	Position	PCR round	Refs
HDV04-F	GGATGCCCAGGTCGGACCG	856–874	1^st^ round PCR	[31]
HDV05-R	AAGAAGAGRAGCCGGCCCGY	1159–1179	1^st^ round PCR	
HDV06-F	ATGCCATGCCGACCCGAAGA	888–907	2^nd^ round PCR	
HDV07-R	GGGGAGCGCCCGGDGGCGG	1104–1122	2^nd^ round PCR	
HDV57-F	GAGAAMYCACCTCCAGAGGA	299–318	1^st^ round PCR	[34]
HDV60-R	TCCCATTCGCCATTACCGA	752–770	1^st^ round PCR	
HDV48-F	AGAGGACCCCTTCAGCGAAC	313–332	2^nd^ round PCR	
HDV54-R	CCGGGATAAGCCTCACTCG	467–485	2^nd^ round PCR	
HBV22-F	TGCTGCTATGCCTCATCTTC	414–433	1^st^ round PCR	[35]
HBV65-R	CAAAGACAAAAGAAAATTGG	822–803	1^st^ round PCR	
HBV66-R	CACAGATAACAAAAAATTGG	822–803	1^st^ round PCR	
HBV24-F	CAAGGTATGTTGCCCGTTTGTCCT	455–478	2^nd^ round PCR	
HBV41-R	GGACTCACGATGCTGTACAG	786–767	2^nd^ round PCR	
HBV64-R	GGACTCAMGATGYTGCACAG	786–767	2^nd^ round PCR	

In order to determine HDV genotypes the L-HDAg (nucleotides 888 to 1122 as referred to NC001653) regions were amplified as previously described [31].Nested PCRs were performed using primer pairs HDV04-F and HDV05-R for first PCR and primer pairs HDV06-F and HDV07-R for second PCR (Table 1). PCR reactions were performed at 95°C for 2 min followed by 95°C for 30 sec, 54°C for 45 sec, and 72°C for 45 sec for 35 cycles, with a final extension for 10 min at 72°C. The amplicons were visualized on 1.5% agarose-gels. In addition, the results were verified and validated in another, independent laboratory at 108 Military Central hospital and reference Centre in Hanoi, Vietnam, which is certified for hepatitis molecular diagnostics. The PCR amplicons were subsequently used for sequencing to determine patient-specific HDV isolates.

### Quantification of HBV and HBV genotyping

Total viral nucleic acids extracted from 200 μL sera were used for HBV quantification and genotyping. HBV quantification was performed using HBV Real-TM Quant Dx (Sacace Biotechnologies, Como, Italy) following the manufacturer´s instructions. HBV genotyping was performed using nested PCR as described [36]. The first round of HBV-specific nested PCR was performed with 2.0 μL total viral nucleic acids (about 400 ng) using FastStart PCR Master Kit (Roche Diagnostics GmbH, Mannheim, Germany), with three primers (HBV22-F, HBV66-R, and HBV65-R) as described in Table 1. The amplicons from first round were used as templates for the second PCR using three nested primers (HBV24-F, HBV64-R, and HBV41-R) as described in Table 1. Initial denaturation was at 95°C for 2 min. The PCR thermal conditions were performed as first round (95°C for 30 sec, 55°C for 30 sec, and 72°C for 30 sec for 35 cycles) and second round (95°C for 30 sec, 50°C for 30 sec, and 72°C for 30 sec for 30 cycles) followed by a final extension for 10 min at 72°C for both PCR rounds. The amplicons were visualized on 1.5% agarose-gels and were subsequently used for sequencing to determine patient-specific HBV genotypes.

### Sequencing and phylogenetic analyses

In order to determine patient-specific HDV isolates, PCR products were gel-eluted using the Gene JET Gel Extraction Kit (ThermoScientific, Lithuania) following the manufacturer’s instructions. 5 μLof eluted PCR amplicons was subjected to cycle sequencing with 1.0 μLof the ABI Prism BigDye terminator cycle sequencing ready reaction kit (Applied Biosystems Inc., Foster city, California, USA) using 0.5 μL of each inner sense and inner antisense primers (HDV48-F/HDV54-R, and HDV06-F/HDV07-R) on a ABI 3500 Automated Genetic Analyser (Applied Biosystems Inc., Foster city, California, USA).Consensus sequences were generated by alignment of both sequenced strands with forward/sense and reverse/antisense primers after validation using BioEdit 9.7 and DNAstar software V7. The phylogenetic tree was reconstructed from nucleotide sequences using the MEGA 7 software [37]. The phylogenetic tree was reconstructed using the Neighbor-Joining tree methodology that utilizes maximum likelihood approach.

For alignment and HDV genotyping, eight prototype HDV-sequences retrieved from the NCBI GenBank were used (HDV-1: AF098261, AJ000558, AY633627, HM046802, NC001653, KF660600, KF660601, KF660602; HDV-2: KF660599, AF104264, AF425645, AY261457, AY261459, AF261460; HDV-3: AB037947, AB037948, AB037949; HDV-4: AF018077, AF209859; HDV-5: AM183326, AM183331, JA417551; HDV-6: AJ584847, AM183332; HDV-7: AM183333, JA417541; HDV-8: AM183327, AM183330).

For alignment and HBV-genotyping, seven HBV-genotype sequences retrieved from the NCBI GenBank were used (HBV genotype A: Z72478, KU605532; HBV genotype B: D00330, AB033554; HBV genotype C: AY040627, X52939; HBV genotype D: KT235604, AY233296; HBV genotype E: X75657, AB032431; HBV genotype F: AB036920, AB036910; HBV genotype G: AB056515, AF405706).

### Statistical analysis

Statistical analysis was performed by using R software (http://www.r-project.org). Categorical data were compared by Fisher´s exact test. Non-parametric data were compared by using the Mann-Whitney U test, with a 2-tailed p-value <0.05 considered to be statistically significant.

## Results

### Baseline characteristics of the HBsAg-positive patients

This cross-sectional study was performed on 250 chronic HBsAg-positive patients in South Central Vietnam. Of the investigated study subjects, 169/250 (68%) were male and 81/250 (32%) were female individuals. The median age of the patients was 36 (11–76 years). The median HBV loads were 4.4 [2.4–9.2 (log10 HBV-DNA)]. The median of serum alanine aminotransferase (ALT), aspartate aminotransferase (AST) levels, and total and direct bilirubin concentrations were 27 IU/L (10–591), 24 IU/L (9–974), 7.8 μmoL/L (2.2–59.1), 3.0 μmoL/L (0.3–40.5), respectively (Table 2).

**Table 2:** Baseline data and clinical characteristics of the HBV patients.

Characteristics	HBV positive (n = 250)	HDV negative (n = 225)	HDV positive (n = 25)
**Age (years)**	36 (11–76)	36 (11–76)	38 (22–62)
**Gender (male/female)**	169/81	154/71	15/10
**ALT (IU/L)**	27 (10–591)	28 (10–591)	22 (10–253)
**AST (IU/L)**	24 (9–974)	23 (10–974)	24 (9–176)
**Total Bilirubin (μmoL/L)**	7.8 (2.2–59.1)	7.7 (2.2–59.1)	8.3 (2.8–27.6)
**Direct Bilirubin (μmoL/L)**	3.0 (0.3–40.5)	2.9 (0.3–40.5)	3.1 (1.2–11.5)
**HBV load (log**_**10**_ **copies/mL)**	4.4 (2.4–9.2)	4.4 (2.4–9.2)	4.7 (2.5–8.7)

IU: international unit; data are given as median with range; ALT: alanine aminotransferase; AST: aspartate aminotransferase. *P* values are presented for comparisons between HDV negativity *vs*. HDV positivity.

### HDV infection in chronic hepatitis B patients

**Fig 1:**
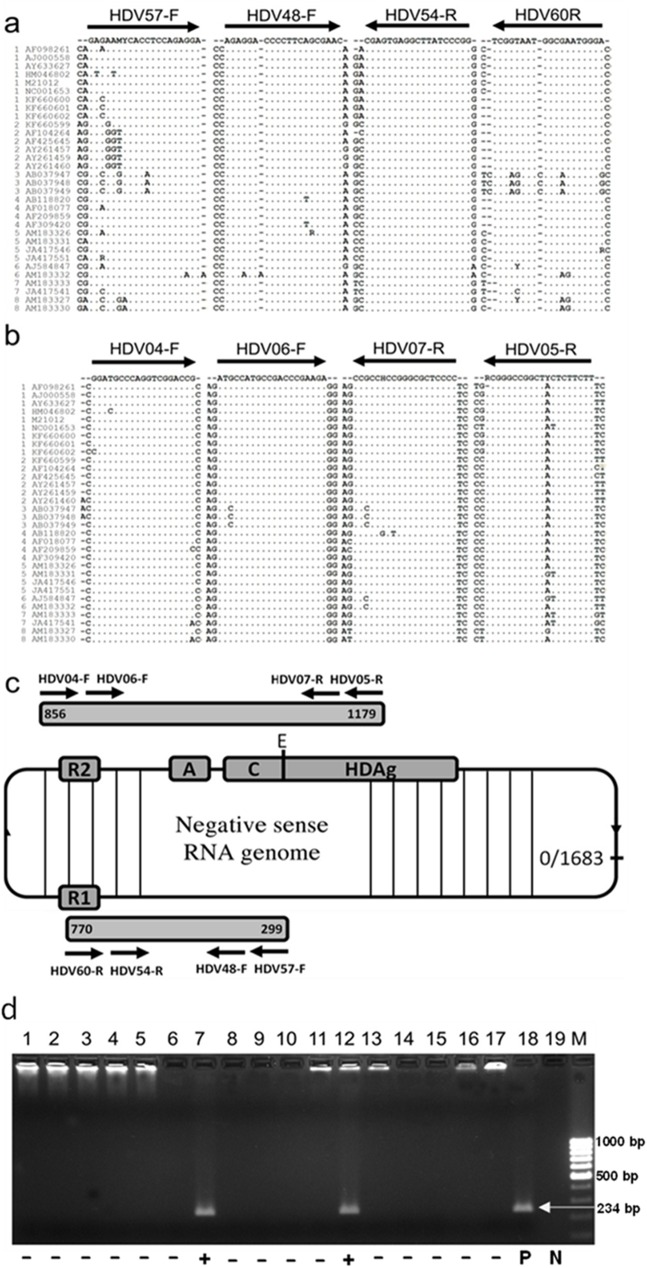
Nested HDV specific RT-PCR. (**a**) Primers for HDV-specific nested PCRs were selected in highly conserved regions of the HDV genome. For detecting HDV RNA genomes, primers HDV57-F and HDV60-R were used for first PCR round, HDV48-F and HDV54-R were used for nested PCR [34]. (**b**) For HDV genotyping, primers HDV04-F and HDV05-R were used for the first round, primers HDV06-F and HDV07-R were used for nested PCR [31]. The primers matched with reference sequences of eight prototype HDV genotypes retrieved from the NCBI-GenBank. The primer sequences target to two different regions of the HDV genome. The numbers 1 to 8 in (a) and (b) of each reference sequence indicate the respective HDV genotypes 1 to 8. (**c**) Schematic representation of the HDV genome and primer binding sites. R1, R2 = ribozyme domain; C = C-terminal amino acid extension; A = PolyA. HDAg: hepatitis delta antigen; E = RNA editing site (position at nt 1015) [38]. Numbering is according to HDV strain NC1001653. (**d**) Representative agarose gel electrophoresis of amplified HDV products from nested-PCR using primers indicated in Fig 1B. The final PCR product length is 234 bp. HDV positive samples were identified in lanes 7 and 12. P, positive control was amplified from a full-length HDV plasmid (lane 18). N, negative control (lane 19). M, marker.

In order to diagnose a HDV infection in chronic HBsAg-positive patients, a recently described sensitive and specific-HDV nested PCR methodology was employed (Fig 1). Alignment of the nested HDV primer sequences showed that the sequences are highly conserved between different HDV-strains and HDV-genotypes of different geographic areas (Fig 1). The HDV nested PCR amplifies a region in the HDV genome which is localized between nucleotides (nt) 313 to nt 485 (numbering is referred to NC001653) that yields an amplicon size of 172 bp (Fig 1).

HDV-RNA was detected in 25/250 (10%) chronic HBsAg-positive patients. Of the 25 HDV positive patients, 15/25 were male (60%) and 10/25 were female (40%). The median age of the HDV-HBV coinfected patients was 38 (22–62 years), and there was no significant difference between males 35 (24–50 years) and females 41.5 (22–62 years) (Table 2).

### Association with HDV infection and HBV genotypes

**Fig 2:**
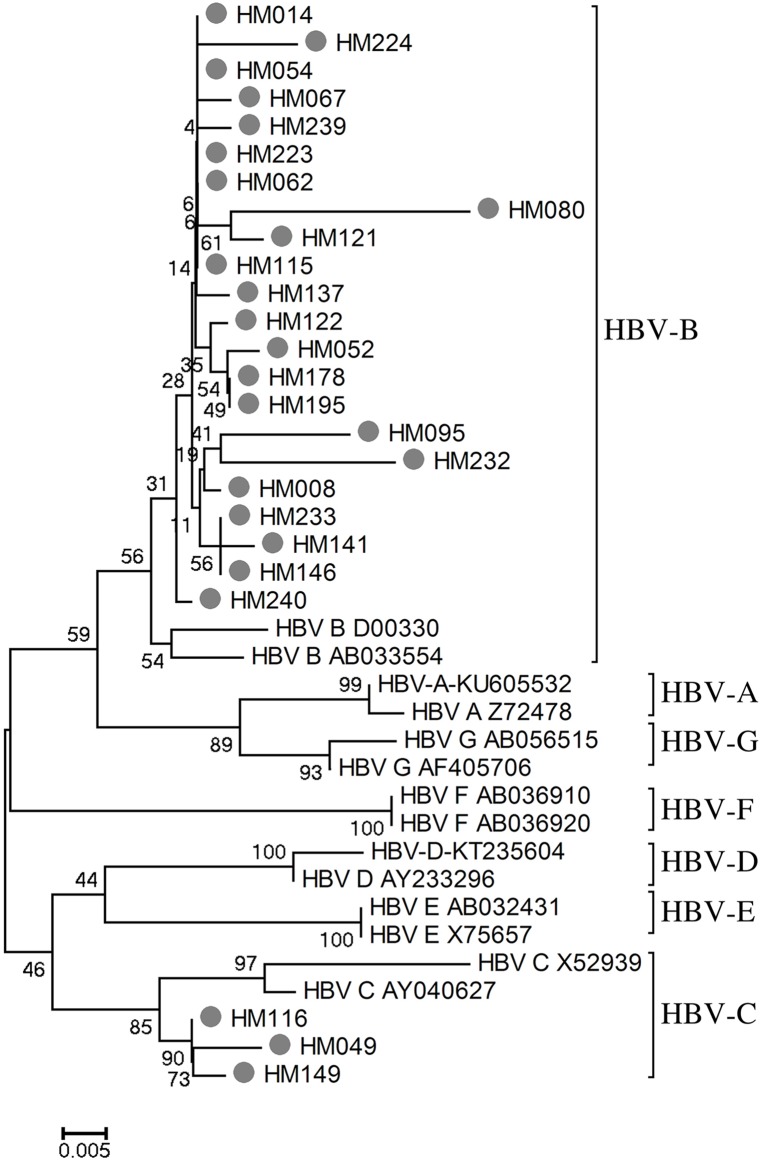
Phylogenetic analyses of HBV genomes in South Central Vietnamese HBsAg-positive patients. Phylogenetic tree was inferred from distance analysis (Kimura 2 parameters model) and neighbor-joining reconstruction from S gene region of HBV sequence (nt 455–786, numbering is referred to HM011485). South Central Vietnamese HBV sequences are referred to as “letter/number”, i.e., “HM115”. The South Central Vietnamese HBV sequences were compared to HBV reference sequences, gathering the seven HBV genotypes (GenBank accession numbers are denoted in the figure). Phylogenetic analysis of HBV region nt455 to nt 786 showed that the HDV sequences were clustered in the Asian HBV-genotype branches 1 and 4.

In order to determine the distribution of HBV genotypes in HDV positive patients, the HDV-HBV coinfected samples were further selected for HBV genotypes. HBV-genotyping showed that HBV-genotypes B (22/25; 88%) and C (3/25; 12%) were predominant (Table 3 and Fig 2). Other HBV genotypes were not detected in this study population. We also compared HBV loads between HBV genotypes within HDV positive samples. HBV loads were not significantly distributed between HBV genotypes (HBV-genotype B *vs*. C: 5.14 ± 2.29 log_10_ copies/mL *vs*. 6.37 ± 1.96 log_10_ copies/mL; *P* = 0.39) (data not shown).

**Table 3:** Distribution of HBV and HDV genotypes in HBV-HDV coinfected individuals.

Nr.	Sample ID	Age	Gender	HBV load^1^ (copies/mL)	HBV genotype	HDV genotype
1	HM008	28	Female	8.7	B	1
2	HM014	22	Female	8.4	B	1
3	HM049	33	Male	8.4	C	1
4	HM052	41	Male	7.7	B	1
5	HM080	62	Female	3.7	B	1
6	HM054	34	Male	4.4	B	2
7	HM062	45	Female	5.7	B	2
8	HM067	45	Male	5.7	B	2
9	HM095	39	Male	3.2	B	2
10	HM115	24	Male	7.9	B	2
11	HM116	24	Male	6.2	C	2
12	HM121	32	Male	3.7	B	2
13	HM122	35	Male	6.0	B	2
14	HM137	31	Male	4.7	B	2
15	HM141	56	Female	6.0	B	2
16	HM146	38	Female	8.7	B	2
17	HM149	39	Male	4.5	C	2
18	HM178	36	Female	8.6	B	2
19	HM195	51	Female	2.9	B	2
20	HM223	26	Female	2.8	B	2
21	HM224	32	Male	2.6	B	2
22	HM232	59	Female	3.7	B	2
23	HM233	48	Male	2.7	B	2
24	HM239	50	Male	2.5	B	2
25	HM240	43	Male	2.8	B	2

^1^values are given as log_10_ copies/mL

### HDV and chronic HBV infection clinical outcomes

**Fig 3:**
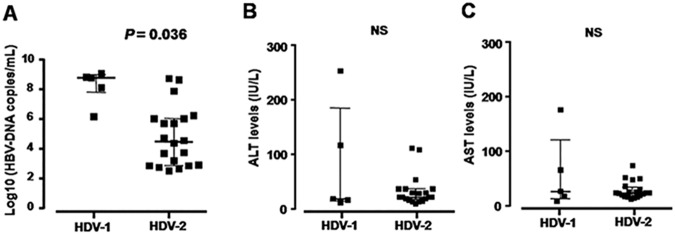
Association with HDV genotypes and clinical parameters in 25 HBV-HDV coinfections. The distribution of HBV-DNA loads (**A**), ALT levels (**B**), and AST levels (**C**) according to HDV genotypes. *P* values were calculated by Mann-Whitney-Wilcoxon test. NS = not significant.

The clinical and subclinical characteristics of the patients with or without HDV infection were presented in Table 2 and Fig 3. The mean age did not differ between two groups (*P* = 0.52). The aminotransferase enzymes (ALT and AST: 43.6 IU/L and 35.0 IU/L *vs*. 43.5 IU/L and 36.3 IU/L, respectively; *P*>0.05) as well as total bilirubin (8.5 μmoL/L *vs*. 8.7 μmoL/L), and direct bilirubin (3.5 μmoL/L *vs*. 3.4 μmoL/L) were not significantly higher in HDV positive compared to HDV negative HBV-infected patients (*P*>0.05). The HBV-DNA loads were not significantly higher in HDV negative patients in comparison to HBV-HDV coinfected patients (5.0 log10 copies/mL *vs*. 5.3 log10 copies/mL; *P* = 0.42).

Although HDV-1 seems to be less predominant in comparison with the HDV-2 in this study, patients with HDV-1 showed higher HBV-DNA loads as compared to those with HDV-2 (median log_10_ (HBV-DNA copies/ml): 8.4 *vs*. 4.5, *P* = 0.036, Fig 3A). Other liver parameters including AST, ALT (Fig 3B and 3C) as well as bilirubin (data not shown) levels were not significantly different between two groups of HDV-1 and HDV-2 patients (*P*>0.05).

### Distribution of HDV genotypes

**Fig 4:**
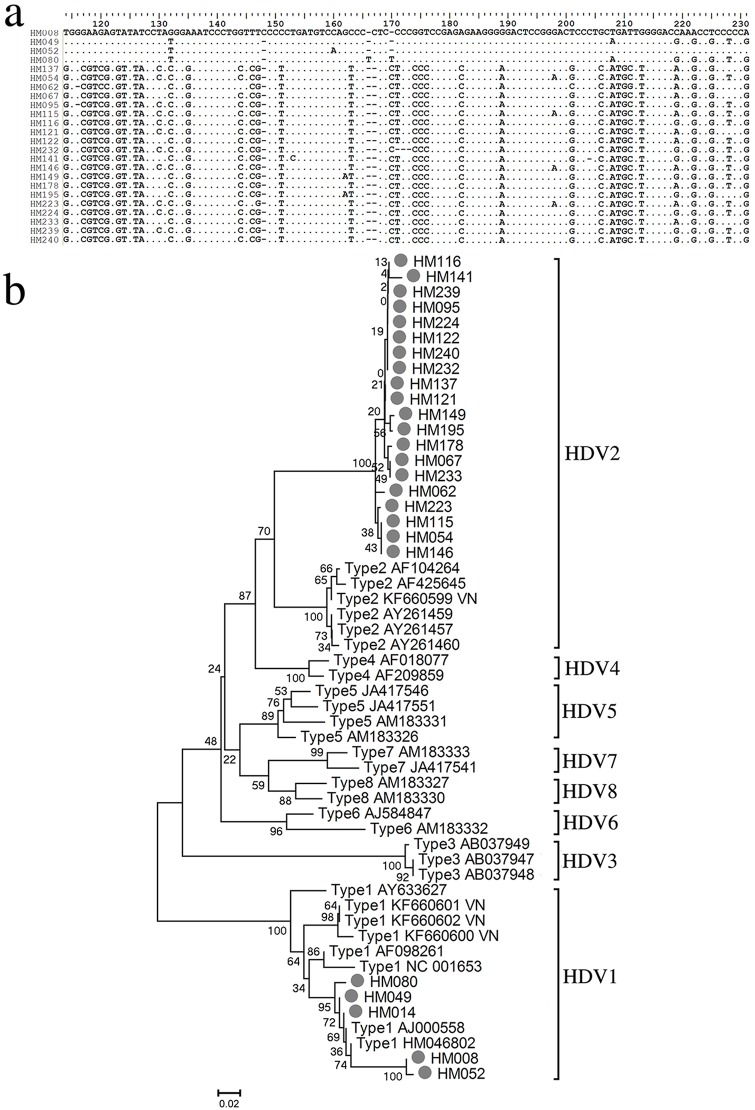
Phylogenetic analyses of HDV genomes in chronic HBsAg-positive patients in South Central Vietnam. (**a**) Representative HDV sequences of South Central Vietnamese HBV-HDV infection patients showing patient-specific HDV isolates. (**b**) Phylogenetic tree was inferred from distance analysis (Kimura 2 parameters model) and neighbor-joining reconstruction from HDV-region sequences. South Central Vietnamese HDV sequences are referred to as “letter/number”, i.e., “HM115”. The South Central Vietnamese HDV sequences were compared to HDV reference sequences, gathering the eight HDV clades (GenBank accession numbers are denoted in the figure). Phylogenetic analysis of HDV region nt 888 to nt 1122 showed that the HDV sequences were clustered in the Asian HDV-branches 1 and 5.

The sequences of the 25 HDV-RNA-positive samples that amplified HDAg are illustrated in Fig 4A. Patient-specific HDV isolates were confirmed by sequence variations of the analysed HDAg gene region (Fig 4B; all sequences were submitted to the GenBank Database and the Accession number allotted from KX897956 to KX897980). Phylogenetic analysis was performed using sequences of the HDV amplicons and analysis showed that all analyzed HDV genotypes belong to either HDV-1 or HDV-2. The HDV strains also clustered in the Asian clade. Notably, 20/25 (80%) HDV isolates fit into the clade of genotype 2 (Fig 4B).

## Discussion

Nearly 40 years after discovery of HDV, the disease caused by this virus yet remains a major problemin regions where HBV is endemic. There are so far no recommended standard therapy and diagnosis in place for HDV treatment in Vietnam. The incidence of HDV and its interaction with HBV carriers is sparsely reported in Central Vietnam, a HBV endemic region. HDV incidences and prevalences are under reported in several Asian countries where HBV is endemic and the significance of HDV infection is poorly studied in Vietnam. This very first study provides epidemiological data on HDV infection in Central Vietnam, with a higher representation of HDV-2 among chronic HBV patients.

In European countries, HDV prevalence has diminished because of available HBV antiviral therapies and due to the successful implementation of HBV vaccination regimen [39]. However, recently, an increasing number of reports claim that HDV incidence is at rise because of increased influx of immigrants from HBV and HDV endemic areas [6, 40] and,therefore, an increased HDV seroprevalence exist (8–20%) [6, 41, 42].In Asia, the HDV infection have been reported in Mongolia (56.5%), Pakistan (≥60%), India (37%), and China (15%) [28, 43–45]. However in other Asian countries such as Korea (0.32%), Indonesia (<0.5%), and Philipines (1.6%), a low HDV prevalence is reported [43, 46, 47].

The hepatitis delta virus distribution varies across different geographical regions and even within a country [6, 14]. For example, the prevalence of HDV infection in HBsAg-positive individuals varied between Western and South Eastern regions of Turkey (5% vs. 27%) [48]. Such intra-regional distributions are likely within a country.In the presented study, we observed that HDV-RNA prevalence is 10% in HBsAg-positive patients compared to a previous study that reported 15% HDV-RNA positive cases among HBsAg cariers in Northern Vietnam [31]. This difference might be due to the region-related burden of the disease or may likely result from the different cohorts of these two studies. In a previous study, hospitalized patients with severe and advanced liver diseases including acute hepatitis, active chronic hepatitis, liver cirrhosis and liver cancer were selected. In this study, we selected HBsAg-positive patients presenting mild hepatitis. HDV was determined as a culprit for more severe hepatitis than HBV monoinfection which can accelerate the progression of chronic hepatitis B to cirrhosis and HCC [49, 50].

Thus far, eight major HDV clades have been identified and distributed over different geographic areas [8]. Overall, HDV-1 is the most common, which is distributed worldwide and has a broad spectrum of pathogenecity [8, 51], whereas HDV-2 to 8 seem to occur more regionally; however, HDV-2 is mainly found in East Asia such as Japan [8, 20]. Although HDV-1 and -2 circulate in Northern Vietnam as reportedearlier, HDV-1 represents a major genotype (HDV-1: 91% *vs*. HDV-2: 9%) [31]. Surprisingly, the results of this current study showed a contradictory pattern of molecular epidemiology with predominance of HDV-2 comapared to HDV-1 (HDV-2: 80% *vs*. HDV-1: 20%). Therefore, we speculate that the distribution of HDV genotypes might differ from region to region as seen in Central Vietnam and Northern Vietnam. However, further studies with a larger samplesizeare needed to confirm this differential distribution of HDV genotypes across regions within Vietnam.

Several studies have concluded that chronic hepatitis D can exacerbate the pre-existing liver damage leading to a rapid progression to liver cirrhosis and liver cancer compared to chronic hepatitis B monoinfection or chronic hepatitis C [1, 3, 4]. Heidrich et al has been demonstrated that, in the clinical course of HBV-HDV coinfection, HBV-DNA levels are frequently low, suggesting inhibitory effects of HDV on HBV regardless of the phase of HBV infection [52]. In addition, among many viral and host factors, HDV genotypes may also contribute to the clinical course and influences the liver disease outcomes. HDV-1 is associated with both severe and mild course, while HDV-2 induces a mild course [14]. Another study investigated the impact of HDV-1 and -2 on clinical implications in HBV-infected patients. The results showed that patients infected with HDV-2 had significantly lower ALT levels and a trend to have lower serum HDV-RNA levels as compared with those infected with HDV-1 [51]. In line with mentioned studies, we showed that the levels of HBV-DNA were higher in HDV-1 patients than in HDV-2 patients. This finding could not reflect fully the clinical significance of HDV towards the clinical outcome of HBV-HDV coinfection due to a relatively small number of HDV positive patients in this cohort. HDV-1 patients were younger than HDV-2 patients in the analyzed patient cohort. Therefore it could not be excluded that the difference in viral loads could be also due to, e.g., an age effect and should be investigated in larger studies.

Although, our current study has characterized the molecular epidemiology of HDV in Central Vietnam, our study has limitations. Firstly, this study is designed as a cross-sectional study and therefore we could not follow up the positve HDV patients over the course of HBV-HDV coinfection. Secondly, we could not assess in detail the influences of HDV infection on the clinical significance and the severity of liver diseases in HBV-HDV coinfection. This might be due to the small sample size and the absence of severe forms of liver diseases, including liver cirrhosis and hepatocellular carcinoma, of the HBsAg-positive outpatients. Importantly, liver biopsies are not available in order to measure the accumulation of the intrahepatic replication intermediates of HBV and HDV.

Our study explored the molecular epidemiology of HDV in Central Vietnam which is to the best of our knowledge the first report with respect to this highly HBV endemic region of Vietnam. Our findings indicate that HDV infection is highly prevalent in which HDV-2 is predominant in Central Vietnam. This study provides new insights into the prevalence and genotype distribution as well as risk of HDV infection in Central Vietnam However, further studies need to be conducted to understand the molecular epidemiology of HDV in different regions in Vietnam.

## Abbreviations

HDV: hepatitis Delta virus
HBV: hepatitis B virus
HBsAg: hepatitis B surface antigens
HDAg: hepatitis D antigens

## References

[1] Fattovich G, Giustina G, Christensen E, Pantalena M, Zagni I, Realdi G, et al (2000) Influence of hepatitis delta virus infection on morbidity and mortality in compensated cirrhosis type B. The European Concerted Action on Viral Hepatitis (Eurohep). Gut. 46(3):420–426. doi: 10.1136/gut.46.3.420 10673308 10.1136/gut.46.3.420 PMC1727859

[2] Shirvani-Dastgerdi E, Tacke F (2015) Molecular interactions between hepatitis B virus and delta virus. World J Virol. 4(2):36–41. doi: 10.5501/wjv.v4.i2.36 25964870 10.5501/wjv.v4.i2.36 PMC4419120

[3] Yurdaydin C, Idilman R, Bozkaya H, Bozdayi AM (2010) Natural history and treatment of chronic delta hepatitis. J Viral Hepat. 17(11):749–756. doi: 10.1111/j.1365-2893.2010.01353.x 20723036 10.1111/j.1365-2893.2010.01353.x

[4] Gish RG, Yi DH, Kane S, Clark M, Mangahas M, Baqai S, et al (2013) Coinfection with hepatitis B and D: epidemiology, prevalence and disease in patients in Northern California. J Gastroenterol Hepatol. 28(9):1521–1525. doi: 10.1111/jgh.12217 23574043 10.1111/jgh.12217

[5] Schweitzer A, Horn J, Mikolajczyk RT, Krause G, Ott JJ (2015) Estimations of worldwide prevalence of chronic hepatitis B virus infection: a systematic review of data published between 1965 and 2013. Lancet. 386(10003):1546–1555. doi: 10.1016/S0140-6736(15)61412-X 26231459 10.1016/S0140-6736(15)61412-X

[6] Wedemeyer H, Manns MP (2010) Epidemiology, pathogenesis and management of hepatitis D: update and challenges ahead. Nat Rev Gastroenterol Hepatol. 7(1):31–40. doi: 10.1038/nrgastro.2009.205 20051970 10.1038/nrgastro.2009.205

[7] Opaleye OO, Japhet OM, Adewumi OM, Omoruyi EC, Akanbi OA, Oluremi AS, et al (2016) Molecular epidemiology of hepatitis D virus circulating in Southwestern Nigeria. Virol J. 13:61 doi: 10.1186/s12985-016-0514-6 27044424 10.1186/s12985-016-0514-6 PMC4820959

[8] Alvarado-Mora MV, Locarnini S, Rizzetto M, Pinho JR (2013) An update on HDV: virology, pathogenesis and treatment. Antivir Ther. 18(3 Pt B):541–548. 23792471 10.3851/IMP2598

[9] Casaca A, Fardilha M, da Cruz e Silva E, Cunha C (2011) The heterogeneous ribonuclear protein C interacts with the hepatitis delta virus small antigen. Virol J. 8:358 doi: 10.1186/1743-422X-8-358 21774814 10.1186/1743-422X-8-358 PMC3160407

[10] Sureau C, Negro F (2016) The hepatitis delta virus: Replication and pathogenesis. J Hepatol. 64(1 Suppl):S102–116. doi: 10.1016/j.jhep.2016.02.013 27084031 10.1016/j.jhep.2016.02.013

[11] Giersch K, Helbig M, Volz T, Allweiss L, Mancke LV, Lohse AW, et al (2014) Persistent hepatitis D virus mono-infection in humanized mice is efficiently converted by hepatitis B virus to a productive co-infection. J Hepatol. 60(3):538–544. doi: 10.1016/j.jhep.2013.11.010 24280293 10.1016/j.jhep.2013.11.010

[12] Chang FL, Chen PJ, Tu SJ, Wang CJ, Chen DS (1991) The large form of hepatitis delta antigen is crucial for assembly of hepatitis delta virus. Proc Natl Acad Sci U S A. 88(19):8490–8494. 1924308 10.1073/pnas.88.19.8490 PMC52534

[13] Pascarella S, Negro F (2011) Hepatitis D virus: an update. Liver Int. 31(1):7–21. doi: 10.1111/j.1478-3231.2010.02320.x 20880077 10.1111/j.1478-3231.2010.02320.x

[14] Hughes SA, Wedemeyer H, Harrison PM (2011) Hepatitis delta virus. Lancet. 378(9785):73–85. doi: 10.1016/S0140-6736(10)61931-9 21511329 10.1016/S0140-6736(10)61931-9

[15] Botelho-Souza LF, Souza Vieira D, de Oliveira Dos Santos A, Cunha Pereira AV, Villalobos-Salcedo JM (2015) Characterization of the Genotypic Profile of Hepatitis Delta Virus: Isolation of HDV Genotype-1 in the Western Amazon Region of Brazil. Intervirology. 58(3):166–171. doi: 10.1159/000431040 26112316 10.1159/000431040

[16] Butt FA, Amin I, Idrees M, Iqbal M (2014) Hepatitis delta virus genotype-1 alone cocirculates with hepatitis B virus genotypes A and D in Pakistan. Eur J Gastroenterol Hepatol. 26(3):319–324. doi: 10.1097/MEG.0000000000000007 24129252 10.1097/MEG.0000000000000007

[17] Bulut Y, Bahcecioglu IH, Aygun C, Oner PD, Ozercan I, Demirdag K (2014) High genetic diversity of hepatitis delta virus in eastern Turkey. J Infect Dev Ctries. 8(1):74–78. doi: 10.3855/jidc.3910 24423715 10.3855/jidc.3910

[18] Abbas Z, Jafri W, Raza S (2010) Hepatitis D: Scenario in the Asia-Pacific region. World J Gastroenterol. 16(5):554–562. doi: 10.3748/wjg.v16.i5.554 20128022 10.3748/wjg.v16.i5.554 PMC2816266

[19] Celik I, Karatayli E, Cevik E, Kabakci SG, Karatayli SC, Dinc B, et al (2011) Complete genome sequences and phylogenetic analysis of hepatitis delta viruses isolated from nine Turkish patients. Arch Virol. 156(12):2215–2220. doi: 10.1007/s00705-011-1120-y 21984217 10.1007/s00705-011-1120-y

[20] Imazeki F, Omata M, Ohto M (1990) Heterogeneity and evolution rates of delta virus RNA sequences. J Virol. 64(11):5594–5599. 2214027 10.1128/jvi.64.11.5594-5599.1990 PMC248612

[21] Lin HH, Lee SS, Yu ML, Chang TT, Su CW, Hu BS, et al (2015) Changing hepatitis D virus epidemiology in a hepatitis B virus endemic area with a national vaccination program. Hepatology. 61(6):1870–1879. doi: 10.1002/hep.27742 25677884 10.1002/hep.27742

[22] Arakawa Y, Moriyama M, Taira M, Hayashi N, Tanaka N, Okubo H, et al (2000) Molecular analysis of hepatitis D virus infection in Miyako Island, a small Japanese island. J Viral Hepat. 7(5):375–381. 10971826 10.1046/j.1365-2893.2000.00244.x

[23] Ivaniushina V, Radjef N, Alexeeva M, Gault E, Semenov S, Salhi M, et al (2001) Hepatitis delta virus genotypes I and II cocirculate in an endemic area of Yakutia, Russia. J Gen Virol. 82(Pt 11):2709–2718. doi: 10.1099/0022-1317-82-11-2709 11602783 10.1099/0022-1317-82-11-2709

[24] di Filippo Villa D, Cortes-Mancera F, Payares E, Montes N, de la Hoz F, Arbelaez MP, et al (2015) Hepatitis D virus and hepatitis B virus infection in Amerindian communities of the Amazonas state, Colombia. Virol J. 12:172 doi: 10.1186/s12985-015-0402-5 26497287 10.1186/s12985-015-0402-5 PMC4619413

[25] Alvarado-Mora MV, Romano CM, Gomes-Gouvea MS, Gutierrez MF, Carrilho FJ, Pinho JR (2011) Dynamics of hepatitis D (delta) virus genotype 3 in the Amazon region of South America. Infect Genet Evol. 11(6):1462–1468. doi: 10.1016/j.meegid.2011.05.020 21645647 10.1016/j.meegid.2011.05.020

[26] Le Gal F, Gault E, Ripault MP, Serpaggi J, Trinchet JC, Gordien E, et al (2006) Eighth major clade for hepatitis delta virus. Emerg Infect Dis. 12(9):1447–1450. doi: 10.3201/eid1209.060112 17073101 10.3201/eid1209.060112 PMC3294742

[27] Andernach IE, Leiss LV, Tarnagda ZS, Tahita MC, Otegbayo JA, Forbi JC, et al (2014) Characterization of hepatitis delta virus in sub-Saharan Africa. J Clin Microbiol. 52(5):1629–1636. doi: 10.1128/JCM.02297-13 24599979 10.1128/JCM.02297-13 PMC3993620

[28] Mumtaz K, Hamid SS, Adil S, Afaq A, Islam M, Abid S, et al (2005) Epidemiology and clinical pattern of hepatitis delta virus infection in Pakistan. J Gastroenterol Hepatol. 20(10):1503–1507. doi: 10.1111/j.1440-1746.2005.03857.x 16174065 10.1111/j.1440-1746.2005.03857.x

[29] Nguyen VT (2012) Hepatitis B infection in Vietnam: current issues and future challenges. Asia Pac J Public Health. 24(2):361–373. doi: 10.1177/1010539510385220 21159700 10.1177/1010539510385220

[30] Hoan NX, Tong HV, Hecht N, Sy BT, Marcinek P, Meyer CG, et al (2015) Hepatitis E Virus Superinfection and Clinical Progression in Hepatitis B Patients. EBioMedicine. 2(12):2080–2086. doi: 10.1016/j.ebiom.2015.11.020 26844288 10.1016/j.ebiom.2015.11.020 PMC4703726

[31] Sy BT, Ratsch BA, Toan NL, Song le H, Wollboldt C, Bryniok A, et al (2013) High prevalence and significance of hepatitis D virus infection among treatment-naive HBsAg-positive patients in Northern Vietnam. PLoS One. 8(10):e78094 doi: 10.1371/journal.pone.0078094 24205106 10.1371/journal.pone.0078094 PMC3799775

[32] Nguyen VT, McLaws ML, Dore GJ (2007) Highly endemic hepatitis B infection in rural Vietnam. J Gastroenterol Hepatol. 22(12):2093–2100. doi: 10.1111/j.1440-1746.2007.05010.x 17645465 10.1111/j.1440-1746.2007.05010.x

[33] Radjef N, Gordien E, Ivaniushina V, Gault E, Anais P, Drugan T, et al (2004) Molecular phylogenetic analyses indicate a wide and ancient radiation of African hepatitis delta virus, suggesting a deltavirus genus of at least seven major clades. J Virol. 78(5):2537–2544. doi: 10.1128/JVI.78.5.2537-2544.2004 14963156 10.1128/JVI.78.5.2537-2544.2004 PMC369207

[34] Sy BT, Nguyen HM, Toan NL, Song LH, Tong HV, Wolboldt C, et al (2014) Identification of a natural intergenotypic recombinant Hepatitis delta virus genotype 1 and 2 in Vietnamese HBsAg-positive patients. Journal of Viral Hepatitis. 22:55–63. doi: 10.1111/jvh.12228 24548489 10.1111/jvh.12228

[35] Al Baqlani SA, Sy BT, Ratsch BA, Al Naamani K, Al Awaidy S, Busaidy SA, et al (2014) Molecular epidemiology and genotyping of hepatitis B virus of HBsAg-positive patients in Oman. PLoS One. 9(5):e97759 doi: 10.1371/journal.pone.0097759 24835494 10.1371/journal.pone.0097759 PMC4023993

[36] Al Baqlani SA, Sy BT, Ratsch BA, Al Naamani K, Al Awaidy S, Al Busaidy S, et al (2014) Molecular epidemiology and genotyping of hepatitis B virus of HBsAg-positive patients in Oman. PLoS ONE. 9:1–10. 10.1371/journal.pone.0097759 PMC4023993 24835494

[37] Kumar S, Stecher G, Tamura K (2016) MEGA7: Molecular Evolutionary Genetics Analysis Version 7.0 for Bigger Datasets. Mol Biol Evol. 33(7):1870–1874. doi: 10.1093/molbev/msw054 27004904 10.1093/molbev/msw054 PMC8210823

[38] Chen R, Linnstaedt SD, Casey JL (2010) RNA editing and its control in hepatitis delta virus replication. Viruses. 2:131–146. doi: 10.3390/v2010131 21994604 10.3390/v2010131 PMC3185552

[39] Gaeta GB, Stroffolini T, Chiaramonte M, Ascione T, Stornaiuolo G, Lobello S, et al (2000) Chronic hepatitis D: a vanishing Disease? An Italian multicenter study. Hepatology. 32(4 Pt 1):824–827. 11003629 10.1053/jhep.2000.17711

[40] Wedemeyer H (2011) Hepatitis D revival. Liver Int. 31 Suppl 1:140–144. 10.1111/j.1478-3231.2010.02408.x 21205152

[41] Cross TJ, Rizzi P, Horner M, Jolly A, Hussain MJ, Smith HM, et al (2008) The increasing prevalence of hepatitis delta virus (HDV) infection in South London. J Med Virol. 80(2):277–282. doi: 10.1002/jmv.21078 18098143 10.1002/jmv.21078

[42] Popescu GA, Otelea D, Gavriliu LC, Neaga E, Popescu C, Paraschiv S, et al (2013) Epidemiology of hepatitis D in patients infected with hepatitis B virus in bucharest: a cross-sectional study. J Med Virol. 85(5):769–774. doi: 10.1002/jmv.23524 23408537 10.1002/jmv.23524

[43] Lu SN, Chen TM, Lee CM, Wang JH, Tung HD, Wu JC (2003) Molecular epidemiological and clinical aspects of hepatitis D virus in a unique triple hepatitis viruses (B, C, D) endemic community in Taiwan. J Med Virol. 70(1):74–80. doi: 10.1002/jmv.10361 12629646 10.1002/jmv.10361

[44] Murhekar MV, Murhekar KM, Arankalle VA, Sehgal SC (2005) Hepatitis delta virus infection among the tribes of the Andaman and Nicobar Islands, India. Trans R Soc Trop Med Hyg. 99(7):483–484. doi: 10.1016/j.trstmh.2004.10.002 15910893 10.1016/j.trstmh.2004.10.002

[45] Tsatsralt-Od B, Takahashi M, Nishizawa T, Endo K, Inoue J, Okamoto H (2005) High prevalence of dual or triple infection of hepatitis B, C, and delta viruses among patients with chronic liver disease in Mongolia. J Med Virol. 77(4):491–499. doi: 10.1002/jmv.20482 16254981 10.1002/jmv.20482

[46] Kim HS, Kim SJ, Park HW, Shin WG, Kim KH, Lee JH, et al (2011) Prevalence and clinical significance of hepatitis D virus co-infection in patients with chronic hepatitis B in Korea. J Med Virol. 83(7):1172–1177. doi: 10.1002/jmv.22095 21541950 10.1002/jmv.22095

[47] Sy NE, Macalagay PS, Paulino GP, Fallarme VD, Reyes RS, Sangalang RP, et al (1990) Serologic classification of acute viral hepatitis at San Lazaro Hospital, Manila, Philippines. Southeast Asian J Trop Med Public Health. 21(1):69–75. 2169652

[48] Degertekin H, Yalcin K, Yakut M, Yurdaydin C (2008) Seropositivity for delta hepatitis in patients with chronic hepatitis B and liver cirrhosis in Turkey: a meta-analysis. Liver Int. 28(4):494–498. doi: 10.1111/j.1478-3231.2008.01673.x 18339076 10.1111/j.1478-3231.2008.01673.x

[49] Rizzetto M, Canese MG, Arico S, Crivelli O, Trepo C, Bonino F, et al (1977) Immunofluorescence detection of new antigen-antibody system (delta/anti-delta) associated to hepatitis B virus in liver and in serum of HBsAg carriers. Gut. 18(12):997–1003. 75123 10.1136/gut.18.12.997 PMC1411847

[50] Uzunalimoglu O, Yurdaydin C, Cetinkaya H, Bozkaya H, Sahin T, Colakoglu S, et al (2001) Risk factors for hepatocellular carcinoma in Turkey. Dig Dis Sci. 46(5):1022–1028. 11341644 10.1023/a:1010705910858

[51] Hsu SC, Syu WJ, Sheen IJ, Liu HT, Jeng KS, Wu JC (2002) Varied assembly and RNA editing efficiencies between genotypes I and II hepatitis D virus and their implications. Hepatology. 35(3):665–672. doi: 10.1053/jhep.2002.31777 11870382 10.1053/jhep.2002.31777

[52] Heidrich B, Serrano BC, Idilman R, Kabacam G, Bremer B, Raupach R, et al (2012) HBeAg-positive hepatitis delta: virological patterns and clinical long-term outcome. Liver Int. 32(9):1415–1425. doi: 10.1111/j.1478-3231.2012.02831.x 22716112 10.1111/j.1478-3231.2012.02831.x

